# The Association of the Pulmonary Artery Pulsatility Index and Right Ventricular Function after Cardiac Surgery

**DOI:** 10.1155/2024/5408008

**Published:** 2024-02-13

**Authors:** Johnny Wei, Abigail Kee, Rachel Dukes, Jack Franke, Vincent Leonardo, Brigid C. Flynn

**Affiliations:** Department of Anesthesiology, University of Kansas Medical Center, Kansas City, KS, USA

## Abstract

**Background:**

The pulmonary artery pulsatility index (PAPi) has been shown to correlate with right ventricular (RV) failure in patients with cardiac disease. However, the association of PAPi with right ventricular function following cardiac surgery is not yet established.

**Methods:**

PAPi and other hemodynamic variables were obtained postoperatively for 959 adult patients undergoing cardiac surgery. The association of post-bypass right ventricular function and other clinical factors to PAPi was evaluated using linear regression. A propensity-score matched cohort for PAPi ≥ 2.00 was used to assess the association of PAPi with postoperative outcomes.

**Results:**

156 patients (16.3%) had post-bypass right ventricular dysfunction defined by visualization on transesophageal echocardiography. There was no difference in postoperative PAPi based on right ventricular function (2.12 vs. 2.00, *p*=0.21). In our matched cohort (*n* = 636), PAPi < 2.00 was associated with increased incidence of acute kidney injury (23.0% vs 13.2%, *p* < 0.01) and ventilator time (6.0 hours vs 5.6 hours, *p*=0.04) but not with 30-day mortality or intensive care unit length of stay.

**Conclusion:**

In a general cohort of patients undergoing cardiac surgery, postoperative PAPi was not associated with postcardiopulmonary bypass right ventricular dysfunction. A postoperative PAPi < 2 may be associated with acute kidney injury.

## 1. Introduction

Right ventricular (RV) dysfunction is associated with poor perioperative outcomes in cardiac surgical patients, and early and accurate diagnosis of this pathology is essential for optimal management to alleviate these associated risks [1–3]. However, continuous monitoring of RV function in the intensive care unit (ICU) presents unique challenges compared to the intraoperative setting, as conventional diagnostic and monitoring modalities have struggled to adequately balance the sensitivity and feasibility of use [4–6].

The pulmonary artery pulsatility index (PAPi) is a novel hemodynamic parameter of right ventricular function gaining momentum in the assessment of cardiac failure. Defined as the ratio of the pulmonary arterial pulse pressure and the right atrial pressure, PAPi can offer rapid insight regarding both the contractility of the right heart and its filling pressures, with a higher PAPi value typically indicating better RV function [7–9]. Consequently, preoperative PAPi has been associated with RV failure following left ventricular assist device (LVAD) implantation and heart transplantation along with other perioperative morbidity such as acute kidney injury [10–13]. Although an optimal range for PAPi has not yet been determined, values less than 2.0 have typically been associated with poor outcomes following cardiac surgery [9, 12–14]. However, the prognostic utility of postoperative PAPi in general cardiac surgical patients as well as its relationship with postoperative RV function is presently unknown.

Thus, the primary aim of this study was to identify the relationship between postoperative PAPi with right ventricular function. The secondary aim was to identify associations between postoperative PAPi and renal injury, mortality, ventilation time, and ICU length of stay.

## 2. Patients and Methods

### 2.1. Patient Data

This was a secondary analysis of a single-center retrospective observational cohort study of adult patients who underwent cardiac surgery between January 1, 2017, and December 31, 2019 [15]. Our study was approved by the institutional review board, and patient consent was waived due to the retrospective nature of our study. Adult patients undergoing cardiac surgical procedures with cardiopulmonary bypass with general endotracheal anesthesia and a pulmonary artery (PA) catheter were included in the study. Exclusion criteria included missing PA hemodynamic data and echocardiographic evaluation of right ventricular function (Supplementary Figure 1). As the original study investigated the association between postoperative PAPi and renal injury, patients who were on hemodialysis preoperatively or those with missing serum creatinine data were also excluded [15]. For duplicate patients, only the index procedure was included in the cohort. The intraoperative anesthetic care and postoperative ICU management of patients were not protocolized but followed routine institutional standard practice [16].

### 2.2. Data Collection

All necessary demographic, clinical, imaging, pharmacologic, and hemodynamic data were acquired from the electronic medical record system (Epic Systems, Verona, WI). Invasive central venous pressure (CVP), PA pressure, mean arterial pressure (MAP), and cardiac index measurements were collected hourly for either the first 48 hours of postoperative care or until PA catheter removal, whichever was shorter, and an average value was obtained. PAPi was calculated as [(PA systolic pressure – PA diastolic pressure)/CVP], where CVP was used as a surrogate for right atrial pressure [7]. RV evaluation from preoperative and intraoperative echocardiograms was recorded and categorized as with or without the presence of dysfunction. Postoperative RV function was defined as postcardiopulmonary bypass transesophageal (TEE) documentation of RV function.

Selective medications administered in the ICU for the duration of their ICU stay were identified and categorized as vasopressors (norepinephrine, vasopressin, and phenylephrine), inotropes (epinephrine, dobutamine, and milrinone), vasodilators (nitroglycerin, nicardipine, and nitroprusside), and diuretics (furosemide, bumetanide). Patients were identified as having received a medication class if it was present on the medication record at any point while they had a PA catheter in place in the ICU.

### 2.3. Outcome

The primary outcome was the association of postoperative PAPi in the ICU and the presence of RV dysfunction defined by post-bypass TEE visualization. Secondary outcomes were association of postoperative PAPi with acute renal injury (defined as a rise in serum creatinine by 50% or more of baseline value) [17], 30-day mortality, total postoperative ventilator hours, and ICU length of stay.

### 2.4. Missing Data

Missing hemodynamic data were imputed using multiple imputations with predictive mean matching in order to maximize statistical inference for our primary outcome. [18] We performed 25 imputations of 10 iterations each. Imputed data were pooled, and a sensitivity analysis was performed by adjusting imputed estimates by 10–20% and repeating the primary outcome analyses to evaluate the robustness of models. Outcome data were not imputed.

### 2.5. Statistical Analysis

Cohort characteristics were described by median and interquartile range for continuous and by frequency for categorical variables. Comparisons between groups were carried out by Student's *t*-test, Wilcoxon rank sum tests, or Fisher's exact test when applicable. A multivariable linear regression model was performed to assess the association between select variables of interest with postoperative PAPi. A natural log transformation for PAPi was performed to improve model performance. Predictor variables with a variance inflation factor of 4 or greater were excluded from the model to adjust for multicollinearity. A Bonferroni–Holm correction was applied to control for multiple comparisons.

For secondary outcomes, we generated a propensity score based on the probability of a postoperative PAPi of 2.0 or greater, estimated by multivariable logistic regression. Variables used to generate propensity scores were based on hypothesized association or confounding with postoperative PAPi and included age, sex, postoperative RV dysfunction, pulmonary disease, ejection fraction, bypass time, surgical procedure, serum creatinine, BMI, and postoperative vasoactive agents (Supplementary Figure 2). Greedy nearest neighbor propensity-score matching without replacement using a prespecified caliper width of 0.1 was used to generate a matched cohort [19]. Baseline differences and outcomes between groups were tested using Student's *t*-test, Wilcoxon rank sum test, or Chi-square when applicable.

All analyses were conducted using R Statistical Software (v4.2.1, R Core Team 2022). A *p* value of <0.05 was used throughout as the threshold for statistical significance.

## 3. Results

A final cohort of 959 patients was included for analysis (Table 1). Of the cohort, 16% (*N* = 156) were identified as having RV dysfunction following cardiopulmonary bypass. Patients with postoperative RV dysfunction were more likely to have a lower baseline left ventricular ejection fraction (40% vs 55%, *p* < 0.01), have a higher baseline preoperative (1.10 vs 0.98 g/dL, *p* < 0.01) and postoperative serum creatinine (1.33 vs 1.10 g/dL, *p* < 0.01), and have preoperative RV dysfunction (39% vs 9%, *p* < 0.01). A higher proportion of patients with RV dysfunction required perioperative use of an intra-aortic balloon pump (15.4% vs 3.4%, *p* < 0.01), underwent urgent or emergent surgery (46.2% vs 37.1%, *p*=0.03), underwent heart transplantation or LVAD implantation (31.5% vs 5.6%, *p* < 0.01), and required postoperative use of inotropes (97% vs 88%, *p* < 0.01) and diuretics (68% vs 34%, *p* < 0.01). Patients with RV dysfunction also maintained their PA catheter for a longer duration (43.7 vs 20.2 hours, *p* < 0.01).

### 3.1. Primary Outcome

**Figure 1:**
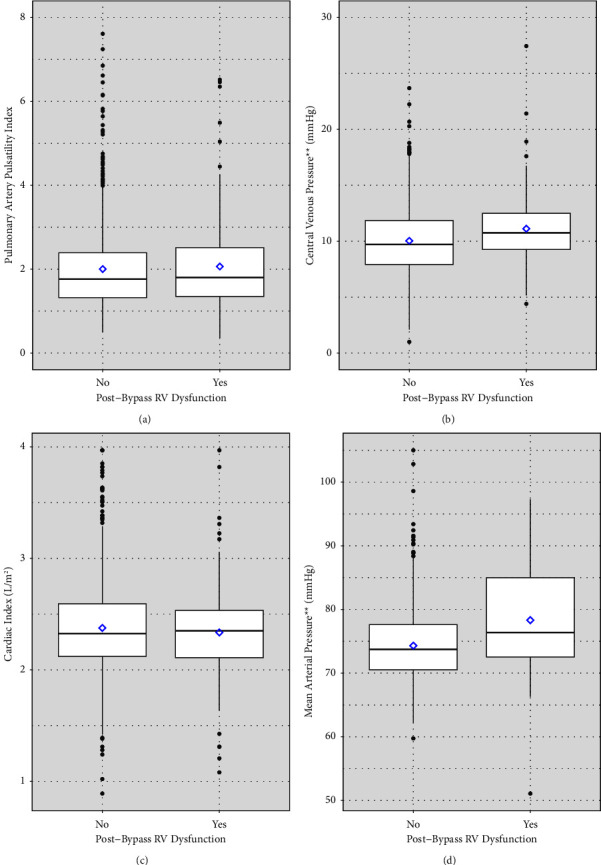
Boxplot of hemodynamic data for patients without and with post-bypass RV dysfunction, respectively. Middle bar represents median, and diamond represents mean values. Median values for each group are as follows: (a) PAPi (1.76 vs 1.81, *p*=0.72); (b) CVP^*∗∗*^ (9.7 vs 10.8 mmHg); (c) CI (2.32 L/m^2^ vs 2.35 L/m^2^); and (d) MAP^*∗∗*^ (73.8 mmHg vs 76.4 mmHg). ^*∗*^*p*<0.05 and ^*∗∗*^*p* < 0.01.

Figure 1 depicts distributions of hemodynamic variables in patients without and with post-bypass RV dysfunction, respectively. Differences in median values for CVP (9.7 vs 10.8 mmHg, *p* < 0.01) and MAP (73.8 mmHg vs 76.4 mmHg, *p* < 0.01) were statistically significant but likely not clinically significant. Differences between median values for PAPi (1.76 vs 1.81, *p*=0.72) and cardiac index (2.32 L/m^2^ vs 2.35 L/m^2^, *p*=0.55) were not statistically significant.

For multivariable regression analysis, surgical status was removed due to a high variance inflation factor of 7.9. Covariates with significant unadjusted associations with PAPi are provided in Table 2 (full regression table provided in Supplementary Table 1). Due to the logarithmic transformation of PAPi, the regression coefficient for each covariate was exponentiated and converted into a geometric mean, which was then reformatted to reflect an arithmetic percent change. Age (0.7%, 95% CI 0.5–0.9, adj *p* < 0.01), preoperative serum creatinine (11%, 95% CI 4.8–17.6, adj *p*=0.01), valvular procedure (9.9%, 95% CI 3.5–16.7, adj *p*=0.04), and CVP (−9.3%, 95% CI −9.9–(−8.6), adj *p* < 0.01) were associated with postoperative PAPi. Post-bypass RV dysfunction was not significantly associated with postoperative PAPi (adj *p*=0.07). The use of inotropes (12.9%, 95% CI 0.8–26.5, adj *p*=0.51) and diuretics (6%, 95% CI 1.0–11.2%) was not associated with postoperative PAPi in adjusted analysis, while the use of vasodilators and vasoconstrictors did not meet significance thresholds in univariate analysis (Supplementary Table 1).

### 3.2. Secondary Outcome

Clinical characteristics of our propensity-matched cohort are given in Table 3. After matching, the cohort included 318 patients in each group. Standardized mean differences of covariates were 0.1 or less, indicating appropriate covariate balancing (Supplementary Figure 2). Patients in the PAPi <2.00 group had a median postoperative PAPi of 1.48 (IQR 1.20–1.71) and postoperative CVP of 10.9 mmHg (IQR 9.4–12.8). Patients in the PAPi < 2.00 group also had a higher incidence of AKI (23.0% vs 13.2%, *p* < 0.01) and marginally increased hours on mechanical ventilation postoperatively (6.0 vs 5.6, *p*=0.04) (Table 4).

### 3.3. Sensitivity Analysis

The cardiac index was missing for 22% (*n* = 208) of the study cohort. The distribution of imputed values overall approximated the distribution of nonmissing values (Supplementary Figure 3). Repeated regression modelling for ln (PAPi) with imputed values adjusted by either 0.24 or 0.48, reflected 10% and 20% of the mean value of the nonmissing index values. Overall, the magnitude of the associations with the outcome of ln (PAPi) remained robust (Supplementary Table 2).

## 4. Discussion

The present study found that in patients undergoing cardiac surgery with cardiopulmonary bypass, there were no differences in postoperative PAPi between patients with and without right ventricular dysfunction on post-bypass TEE. Furthermore, in a propensity-matched cohort, patients with a postoperative PAPi of less than 2.00 had a higher incidence of AKI and slightly longer, but less clinically significant, ventilation time compared to patients with PAPi of 2.00 or greater.

With known challenges in identifying RV dysfunction, PAPi was conceptualized to offer a simplified but unique assessment of right ventricular function [8–10, 20]. PAPi not only reflects RV contractility but may be more sensitive to changing RV loading conditions than other hemodynamic measurements [8]. Following its initial use in patients with right ventricular myocardial infarction, it has been associated with clinical outcomes in diverse patient populations, most notably in patients with heart failure [10–12, 20–22]. However, current studies on PAPi emphasize the association of preoperative PAPi and postoperative clinical outcomes rather than identifying validated postoperative PAPi thresholds.

This study illustrates that PAPi is not a robust surrogate for RV function in a diverse postoperative cardiac surgical patient cohort. This could be because PAPi is not as sensitive of a marker of RV dysfunction in the setting of rapid hemodynamic and volume changes following cardiac surgery [15, 23, 24]. Indeed, RV failure in the perioperative cardiac surgical period is multifactorial and affected by preload, afterload, and stunning from perioperative insults such as cardiopulmonary bypass [25–28]. Alternatively, our findings could be confounded by awareness of the TEE assessment of RV dysfunction which could have led the ICU team to modify management in order to initiate or enhance RV support [25, 28, 29]. This would lead to improved PAPi, theoretically, and negate the association of post-bypass RV dysfunction with lower PAPi values. However, there was no independent association identified between vasoactive or diuretic medication exposure in the ICU and postoperative PAPi. This could simply be because the majority of patients in this study received RV support medications, as is common in postoperative management in the present era [5, 25, 28, 29]. Thus, the impact of vasoactive medications on RV dysfunction and correlation to postoperative PAPi cannot be fully deduced by the present study.

Additionally, this study and those investigating postoperative PAPi illustrate that PAPi values need to be contextualized for various patient populations. We selected a postoperative PAPi threshold of 2.00 based on available current literature that identified similar values as a threshold for perioperative outcomes [9, 11–14, 22]. Although the present study found an association of this threshold with perioperative morbidity such as AKI, this finding may be driven by CVP [15, 30, 31]. A doubling of CVP will halve the PAPi value, which would have major implications if postoperative management is based on PAPi values alone. Ultimately, studies on postoperative PAPi values of “normalcy” need to be individualized for various patient populations, especially in the setting of complex physiology such as pulmonary hypertension, heart transplantation, or mechanical circulatory support [8, 23, 24].

### 4.1. Limitations

There are a number of important limitations of our study. We did not evaluate prospective development of RV dysfunction in our patients and rather relied on intraoperative post-bypass evaluation of RV function. This could have led to the initiation of therapeutics that significantly affect postoperative PAPi values. However, this mimics current practice in ICU management of RV dysfunction where there is limited accurate monitoring of this morbidity. Additionally, data acquisition of medications did not account for the frequency or total dosing of each class of medication, but only for the initiation and use of the medication. Lastly, discrepancies in qualitative TEE assessment of RV function in cardiac surgical patients may be present, especially when most of these assessments rely upon sonographer proficiency and judgement [32]. However, the definition of RV dysfunction as ascertained from the official TEE report mirrors real-world practice in how RV failure is identified in cardiac surgical patients and is generally thought to be the gold standard for RV failure identification in these patients [25].

## 5. Conclusion

Post-bypass RV dysfunction was not associated with postoperative PAPi in the ICU in a mixed cardiac surgical population. When adjusted for confounders, a postoperative PAPi less than 2.00 was associated with an increased incidence of AKI and a less clinically significant increase in ventilator time but was not associated with 30-day mortality and ICU length of stay. Overall, our study emphasizes the importance of developing an accurate and continuous assessment of RV function following cardiac surgery and further validating postoperative PAPi values for individual patient populations.

## Figures and Tables

**Table 1:** Cohort characteristics.

	Total *N* = 959	Post-bypass RV dysfunction		
		No *N* = 803	Yes *N* = 156	*p* value
Age (years)	64.0 [56.0, 71.0]	64.0 [56.0, 71.0]	62.0 [53.8, 70.3]	0.03
Male sex (%)	680 (70.9)	578 (72.0)	102 (65.4)	0.12
Chronic lung disease (%)	210 (21.9)	167 (20.8)	43 (27.6)	0.08
BMI (kg/m^2^)	28.9 [25.8, 33.0]	28.8 [25.8, 32.7]	29.1 [25.5, 34.5]	0.52
Ejection fraction (%)	55.0 [43.5, 60.0]	55.0 [50.0, 60.0]	40.0 [20.0, 55.0]	<0.01
Bypass time (min)	101 [77, 131]	101 [78, 130]	101 [69, 131]	0.46
Preop serum creatinine (g/dL)	1.00 [0.84, 1.19]	0.98 [0.84, 1.17]	1.10 [0.89, 1.31]	<0.01
Postop serum creatinine (g/dL)	1.14 [0.90, 1.51]	1.10 [0.90, 1.48]	1.33 [1.10, 2.00]	<0.01
IABP use (%)	51 (5.3)	27 (3.4)	24 (15.4)	<0.01
Surgical status (%)				
Elective	589 (61.4)	505 (62.9)	84 (53.8)	0.03
Urgent	310 (32.3)	254 (31.6)	56 (35.9)	
Emergent	60 (6.3)	44 (5.5)	16 (10.3)	
Procedure (%)				
CABG	429 (44.7)	383 (47.7)	46 (29.5)	<0.01
Valve	157 (16.4)	139 (17.3)	18 (11.5)	
Aortic	130 (13.6)	113 (14.1)	17 (10.9)	
CABG & valve	71 (7.4)	58 (7.2)	13 (8.3)	
OHT	34 (3.5)	23 (2.9)	11 (7.1)	
LVAD	60 (6.3)	22 (2.7)	38 (24.4)	
Other^*∗*^	78 (8.1)	65 (8.1)	13 (8.3)	
Preop RV dysfunction (%)	134 (14.0)	73 (9.1)	61 (39.1)	<0.01
Vasopressor use (%)	824 (85.9)	682 (84.9)	142 (91.0)	0.06
Inotrope use (%)	856 (89.3)	705 (87.8)	151 (96.8)	<0.01
Vasodilator use (%)	732 (76.3)	635 (79.1)	97 (62.2)	<0.01
Diuretic use (%)	380 (39.6)	274 (34.1)	106 (67.9)	<0.01
PA catheter duration (hours)	20.7 [17.7, 38.8]	20.2 [17.4, 25.8]	43.7 [22.3, 47.6]	<0.01

Continuous data presented as median [interquartile range] and categorical data presented as frequency (percentage of total). RV = right ventricle; BMI = body mass index; IABP = intra-aortic balloon pump; CABG = coronary artery bypass graft; OHT = orthotopic heart transplantation; LVAD = left ventricular assist device; PA = pulmonary artery. ^*∗*^Other = pulmonary thrombectomy, cardiac tumor removal, subaortic membrane removal.

**Table 2:** Multivariable linear regression analysis.

	% ΔPAPi (95% C.I.)	*p* (unadj)	*p* (adj)
(Intercept)	157.2 (66.7–296.8)	<0.01	<0.01
Age (years)	0.7 (0.5–0.9)	<0.01	<0.01
Lung disease	7.7 (2.4–13.2)	<0.01	0.07
BMI (kg/m^2^)	0.4 (0.1–0.8)	0.02	0.30
Preop serum creatinine (g/dL)	11 (4.8–17.6)	<0.01	0.01
Surgical procedure			
CABG (reference)	N/A	N/A	N/A
Valvular	9.9 (3.5–16.7)	<0.01	0.04
CABG and valve	11.1 (2–20.9)	0.02	0.26
Post-bypass RV dysfunction	9.5 (3–16.3)	<0.01	0.07
Cardiac index (L/m^2^)	5.9 (0.4–11.8)	0.04	0.51
Central venous pressure (mmHg)	−9.3 (−9.9–−8.6)	<0.01	<0.01
Inotrope use	12.9 (0.8–26.5)	0.04	0.51
Diuretic use	6 (1–11.2)	0.02	0.30

Linear regression table performed ln(PAPi) as the dependent variable. Beta coefficient for each predictor was exponentiated to geometric mean difference (odds ratio) per unit change in predictor. Geometric mean adjusted to reflect percent change in PAPi per unit change in covariate. Adjusted *p* values are based on Bonferroni–Holm correction.

**Table 3:** Propensity-matched cohort for PAPi ≥ 2.0.

	PAPi < 2.0 *N* = 318	PAPi ≥ 2.0 *N* = 318	*p* value
Age (years)	66.00 [59.00, 72.00]	67.00 [58.00, 72.00]	0.82
Male sex (%)	237 (74.5)	244 (76.7)	0.58
Chronic lung disease (%)	76 (23.9)	74 (23.3)	0.93
BMI (kg/m^2^)	28.7 [25.8, 32.1]	28.1 [24.9, 32.2]	0.16
Ejection fraction (%)	55.00 [43.00, 60.00]	55.00 [43.50, 60.00]	0.91
Bypass time (min)	101.00 [77.00, 130.00]	101.00 [78.00, 129.00]	0.94
Preop serum creatinine (g/dL)	1.00 [0.85, 1.20]	1.01 [0.87, 1.18]	0.84
Postop serum creatinine (g/dL)	1.16 [0.98, 1.56]	1.11 [0.92, 1.50]	0.25
IABP use (%)	14 (4.4)	13 (4.1)	0.99
Surgical status (%)			
Elective	195 (61.3)	205 (64.5)	0.27
Urgent	110 (34.6)	94 (29.6)	
Emergent	13 (4.1)	19 (6.0)	
Procedure (%)			
CABG	142 (44.7)	148 (46.5)	0.96
Valve	62 (19.5)	60 (18.9)	
Aortic	38 (11.9)	39 (12.3)	
CABG and valve	30 (9.4)	24 (7.5)	
OHT	6 (1.9)	9 (2.8)	
LVAD	21 (6.6)	17 (5.3)	
Other	21 (6.6)	21 (6.6)	
Preop RV dysfunction (%)	42 (13.2)	48 (15.1)	0.51
Postop RV dysfunction (%)	51 (16.0)	52 (16.4)	0.99
Vasopressor use (%)	269 (84.6)	272 (85.5)	0.82
Inotrope use (%)	279 (87.7)	282 (88.7)	0.81
Vasodilator use (%)	252 (79.2)	245 (77.0)	0.66
Diuretic use (%)	105 (33.0)	105 (33.0)	0.90
PA catheter duration (hours)	20.6 [17.5, 32.9]	20.5 [17.70, 33.4]	0.99

Continuous data presented as median [interquartile range], and categorical data presented as count (percentage of total).

**Table 4:** Secondary outcomes in the propensity-matched cohort for PAPi ≥ 2.00.

	PAPi < 2.00 *N* = 318	PAPi ≥ 2.00 *N* = 318	*p* value
Hemodynamics			
PAPi	1.48 (1.20, 1.71)	2.67 (2.27, 3.28)	<0.01
CVP (mmHg)	10.9 (9.4, 12.8)	8.1 (6.5, 9.6)	<0.01
Cardiac index (L/m^2^)	2.27 (2.09, 2.50)	2.36 (2.14, 2.64)	<0.01
MAP (mmHg)	74.4 (71.4, 78.1)	72.8 (69.8, 76.8)	<0.01
Clinical outcomes			
Acute kidney injury (%)	73 (23.0)	42 (13.2)	<0.01
30-day mortality (%)	7 (2.2)	7 (2.2)	0.99
Ventilator time (hours)	6.0 (4.5, 13.5)	5.6 (4.4, 9.8)	0.04
ICU length of stay (hours)	48.2 (23.7, 89.5)	45.0 (23.6, 73.2)	0.20

Continuous data presented as median (interquartile range), and categorical data presented as count (percentage of total).

## Data Availability

The data used to support the findings of this study are available from the corresponding author upon request.

## Abbreviations

AKI: Acute kidney injury
BMI: Body mass index
CABG: Coronary artery bypass graft
CI: Cardiac index
CVP: Central venous pressure
IABP: Intra-aortic balloon pump
ICU: Intensive care unit
LVAD: Left ventricular assist device
MAP: Mean arterial pressure
OHT: Orthotopic heart transplantation
PA: Pulmonary artery
PAPi: Pulmonary artery pulsatility index
RV: Right ventricular/right ventricle
TEE: Transesophageal echo
TTE: Transthoracic echo.

## Supplementary Materials

Supplementary Materials Supplementary Figure 1: flowchart of study design with reasons for exclusion. Supplementary Figure 2: covariate balance after propensity-score matching for PAPi > 2.0 using selected variables. Dark bars represent a standardized mean difference (SMD) of 0.2 and dashed bars represent an SMD of 0.1. Supplementary Figure 3: strip plot depicting the distribution of nonmissing values for cardiac index (blue) with imputed values (red). 25 imputations of 10 iterations each were performed. The final estimates used were pooled from imputed values. Supplementary Table 1: multivariable regression table with % ΔPAPi as an outcome of interest, with all included predictor variables. Supplementary Table 2: sensitivity analysis of multivariable regression analysis using imputed values for hemodynamic data and imputed values adjusted by ±10% and ±20% of nonmissing values for cardiac index.
