# Supplementary material for: The Association of the Pulmonary Artery Pulsatility Index and Right Ventricular Function after Cardiac Surgery
Source: Crit Care Res Pract. 2024 Feb 13;2024:5408008. doi: 10.1155/2024/5408008 (PMC10878756; doi:10.1155/2024/5408008)
Supplement: Supplementary Materials — Supplementary Figure 1: flowchart of study design with reasons for exclusion. Supplementary Figure 2: covariate balance after propensity-score matching for PAPi > 2.0 using selected variables. Dark bars represent a standardized mean difference (SMD) of 0.2 and dashed bars represent an SMD of 0.1. Supplementary Figure 3: strip plot depicting the distribution of nonmissing values for cardiac index (blue) with imputed values (red). 25 imputations of 10 iterations each were performed. The final estimates used were pooled from imputed values. Supplementary Table 1: multivariable regression table with % ΔPAPi as an outcome of interest, with all included predictor variables. Supplementary Table 2: sensitivity analysis of multivariable regression analysis using imputed values for hemodynamic data and imputed values adjusted by ±10% and ±20% of nonmissing values for cardiac index. [file 5408008.f1.zip › Supplementary Tables.docx]

Supplementary Table 1: Multivariable Regression

|  | Coefficient | % ΔPAPi (95% C.I.) | P (unadj) | P (adj) |
| --- | --- | --- | --- | --- |
| (Intercept) | 0.945 | 157.2 (66.7 - 296.8) | < 0.01 | < 0.01 |
| Age (years) | 0.007 | 0.7 (0.5 - 0.9) | < 0.01 | < 0.01 |
| Sex (Male) | 0.026 | 2.6 (-2.2 - 7.7) | 0.29 | > 0.99 |
| Lung Disease | 0.074 | 7.7 (2.4 - 13.2) | < 0.01 | 0.07 |
| BMI (kg/m^2^) | 0.004 | 0.4 (0.1 - 0.8) | 0.02 | 0.30 |
| Ejection Fraction (%) | 0.000 | 0 (-0.2 - 0.2) | 0.86 | > 0.99 |
| Bypass Time (min) | 0.000 | 0 (-0.1 - 0.1) | 0.91 | > 0.99 |
| Preop Serum Creatinine (g/dL) | 0.105 | 11 (4.8 - 17.6) | < 0.01 | 0.01 |
| IABP Use | -0.026 | -2.6 (-11.4 - 7.2) | 0.60 | > 0.99 |
| Surgical Procedure |  |  |  |  |
| CABG (baseline) | N/A | N/A | N/A | N/A |
| Valvular | 0.094 | 9.9 (3.5 - 16.7) | < 0.01 | 0.04 |
| Aortic | 0.052 | 5.3 (-1.7 - 12.8) | 0.14 | > 0.99 |
| CABG & Valve | 0.105 | 11.1 (2 - 20.9) | 0.02 | 0.26 |
| OHT | -0.046 | -4.5 (-15.6 - 8) | 0.46 | > 0.99 |
| LVAD | 0.040 | 4.1 (-7.5 - 17.1) | 0.50 | > 0.99 |
| Other | 0.065 | 6.7 (-1.3 - 15.4) | 0.10 | > 0.99 |
| Preop RV Dysfunction | 0.043 | 4.3 (-2.3 - 11.5) | 0.21 | > 0.99 |
| Post-Bypass RV Dysfunction | 0.090 | 9.5 (3 - 16.3) | < 0.01 | 0.07 |
| Mean Arterial Pressure (mmHg) | -0.004 | -0.4 (-0.8 - 0) | 0.06 | 0.67 |
| Cardiac Index (L/m^2^) | 0.058 | 5.9 (0.4 - 11.8) | 0.04 | 0.51 |
| Central Venous Pressure (mmHg) | -0.097 | -9.3 (-9.9 - -8.6) | < 0.01 | < 0.01 |
| Inotrope Use | 0.121 | 12.9 (0.8 - 26.5) | 0.04 | 0.51 |
| Vasodilator Use | -0.031 | -3.1 (-7.8 - 1.8) | 0.22 | > 0.99 |
| Diuretic Use | 0.058 | 6 (1 - 11.2) | 0.02 | 0.30 |
| Vasopressor Use | -0.057 | -5.6 (-14.6 - 4.5) | 0.27 | > 0.99 |

*Linear regression table with ln(PAPi) as the dependent variable. Regression coefficient represents change in ln(PAPi) per unit change in variable. % ΔPAPi represents exponent of coefficient and indicates % change in PAPi per unit change in variable. P-values are based on regression coefficient. Adjusted P-values are based on Bonferroni-Holm correction. Abbreviations: PAPi = pulmonary artery pulsatility index; BMI = body mass index; IABP = intra-aortic balloon pump; CABG = coronary artery bypass graft; OHT = orthotopic heart transplantation; LVAD = left ventricular assist device; PA = pulmonary artery*

Supplementary Table 2: Sensitivity Analysis

|  | Imputed | | Imputed – 0.24 | | Imputed – 0.48 | | Imputed + 0.24 | | Imputed – 0.48 | | Non-Missing | |
| --- | --- | --- | --- | --- | --- | --- | --- | --- | --- | --- | --- | --- |
|  | β | P value | β | P value | β | P value | β | P value | β | P value | β | P value |
| (Intercept) | 0.945 | < 0.01 | 0.905 | < 0.01 | 0.897 | < 0.01 | 1.009 | < 0.01 | 1.078 | < 0.01 | 1.066 | < 0.01 |
| Age (years) | 0.007 | < 0.01 | 0.007 | < 0.01 | 0.007 | < 0.01 | 0.006 | < 0.01 | 0.006 | < 0.01 | 0.006 | < 0.01 |
| Male Sex (%) | 0.026 | 0.29 | 0.024 | 0.34 | 0.023 | 0.35 | 0.029 | 0.24 | 0.032 | 0.19 | 0.016 | 0.56 |
| Lung Disease (%) | 0.074 | < 0.01 | 0.074 | < 0.01 | 0.073 | < 0.01 | 0.074 | < 0.01 | 0.073 | < 0.01 | 0.071 | 0.01 |
| BMI (kg/m^2^) | 0.004 | 0.02 | 0.004 | 0.02 | 0.004 | 0.02 | 0.004 | 0.02 | 0.004 | 0.02 | 0.005 | 0.01 |
| Ejection Fraction (%) | < 0.001 | 0.86 | < 0.001 | 0.82 | < 0.001 | 0.79 | < 0.001 | 0.89 | < 0.001 | 0.92 | < 0.001 | 0.62 |
| Bypass Time (min) | < 0.001 | 0.91 | < 0.001 | 0.90 | < 0.001 | 0.87 | < 0.001 | 0.90 | < 0.001 | 0.88 | < 0.001 | 0.89 |
| Preop Serum Creatinine (g/dL) | 0.105 | < 0.01 | 0.104 | < 0.01 | 0.103 | < 0.01 | 0.105 | < 0.01 | 0.105 | < 0.01 | 0.092 | < 0.01 |
| IABP Use (%) | -0.026 | 0.59 | -0.025 | 0.61 | -0.025 | 0.61 | -0.027 | 0.58 | -0.028 | 0.57 | -0.012 | 0.86 |
| Surgical Procedure (%) |  |  |  |  |  |  |  |  |  |  |  |  |
| CABG | NA | NA | NA | NA | NA | NA | NA | NA | NA | NA | NA | NA |
| Valve | 0.094 | < 0.01 | 0.092 | < 0.01 | 0.090 | < 0.01 | 0.097 | < 0.01 | 0.099 | < 0.01 | 0.087 | 0.01 |
| Aortic | 0.052 | 0.14 | 0.050 | 0.16 | 0.048 | 0.17 | 0.054 | 0.13 | 0.055 | 0.12 | 0.032 | 0.38 |
| CABG & Valve | 0.105 | 0.02 | 0.105 | 0.01 | 0.106 | 0.01 | 0.105 | 0.02 | 0.106 | 0.01 | 0.110 | 0.02 |
| OHT | -0.046 | 0.46 | -0.042 | 0.50 | -0.037 | 0.55 | -0.048 | 0.45 | -0.047 | 0.45 | -0.123 | 0.16 |
| LVAD | 0.040 | 0.50 | 0.047 | 0.43 | 0.057 | 0.34 | 0.039 | 0.52 | 0.043 | 0.48 | 0.074 | 0.74 |
| Other | 0.065 | 0.10 | 0.065 | 0.10 | 0.064 | 0.11 | 0.065 | 0.10 | 0.066 | 0.10 | 0.041 | 0.33 |
| Preop RV Dysfunction (%) | 0.043 | 0.21 | 0.042 | 0.21 | 0.042 | 0.21 | 0.043 | 0.20 | 0.043 | 0.21 | 0.010 | 0.80 |
| Post-Bypass RV Dysfunction (%) | 0.090 | < 0.01 | 0.090 | < 0.01 | 0.090 | < 0.01 | 0.090 | < 0.01 | 0.090 | < 0.01 | 0.114 | 0.00 |
| Mean Arterial Pressure (mmHg) | -0.004 | 0.06 | -0.004 | 0.07 | -0.003 | 0.08 | -0.004 | 0.04 | -0.004 | 0.04 | -0.005 | 0.04 |
| Cardiac Index (L/m^2^) | 0.058 | 0.04 | 0.068 | 0.01 | 0.070 | < 0.01 | 0.042 | 0.12 | 0.025 | 0.34 | 0.079 | 0.01 |
| Central Venous Pressure (mmHg) | -0.097 | < 0.01 | -0.097 | < 0.01 | -0.097 | < 0.01 | -0.098 | < 0.01 | -0.098 | < 0.01 | -0.099 | < 0.01 |
| Inotrope Use (%) | 0.121 | 0.04 | 0.122 | 0.03 | 0.123 | 0.03 | 0.119 | 0.04 | 0.118 | 0.04 | 0.084 | 0.18 |
| Vasodilator Use (%) | -0.031 | 0.22 | -0.032 | 0.21 | -0.033 | 0.19 | -0.031 | 0.22 | -0.032 | 0.21 | -0.039 | 0.18 |
| Diuretic Use (%) | 0.058 | 0.02 | 0.059 | 0.02 | 0.060 | 0.02 | 0.058 | 0.02 | 0.058 | 0.02 | 0.052 | 0.05 |
| Vasopressor Use (%) | -0.057 | 0.27 | -0.056 | 0.27 | -0.056 | 0.28 | -0.058 | 0.26 | -0.059 | 0.25 | -0.034 | 0.55 |

*Sensitivity analysis of regression model (Table 2) following data imputation. β reflects regression coefficients using imputed dataset. Regression model was repeated after adjusting imputed values by 0.24 or 0.48, which reflects 10% or 20% of the mean of non-missing cardiac index values, respectively. Regression coefficients obtained with non-missing data are at the end of the table. Abbreviations: PAPi = pulmonary artery pulsatility index; BMI = body mass index; IABP = intra-aortic balloon pump; CABG = coronary artery bypass graft; OHT = orthotopic heart transplantation; LVAD = left ventricular assist device*
