# Supplementary figures and images for: The Association of the Pulmonary Artery Pulsatility Index and Right Ventricular Function after Cardiac Surgery
Source: Crit Care Res Pract. 2024 Feb 13;2024:5408008. doi: 10.1155/2024/5408008 (PMC10878756; doi:10.1155/2024/5408008)

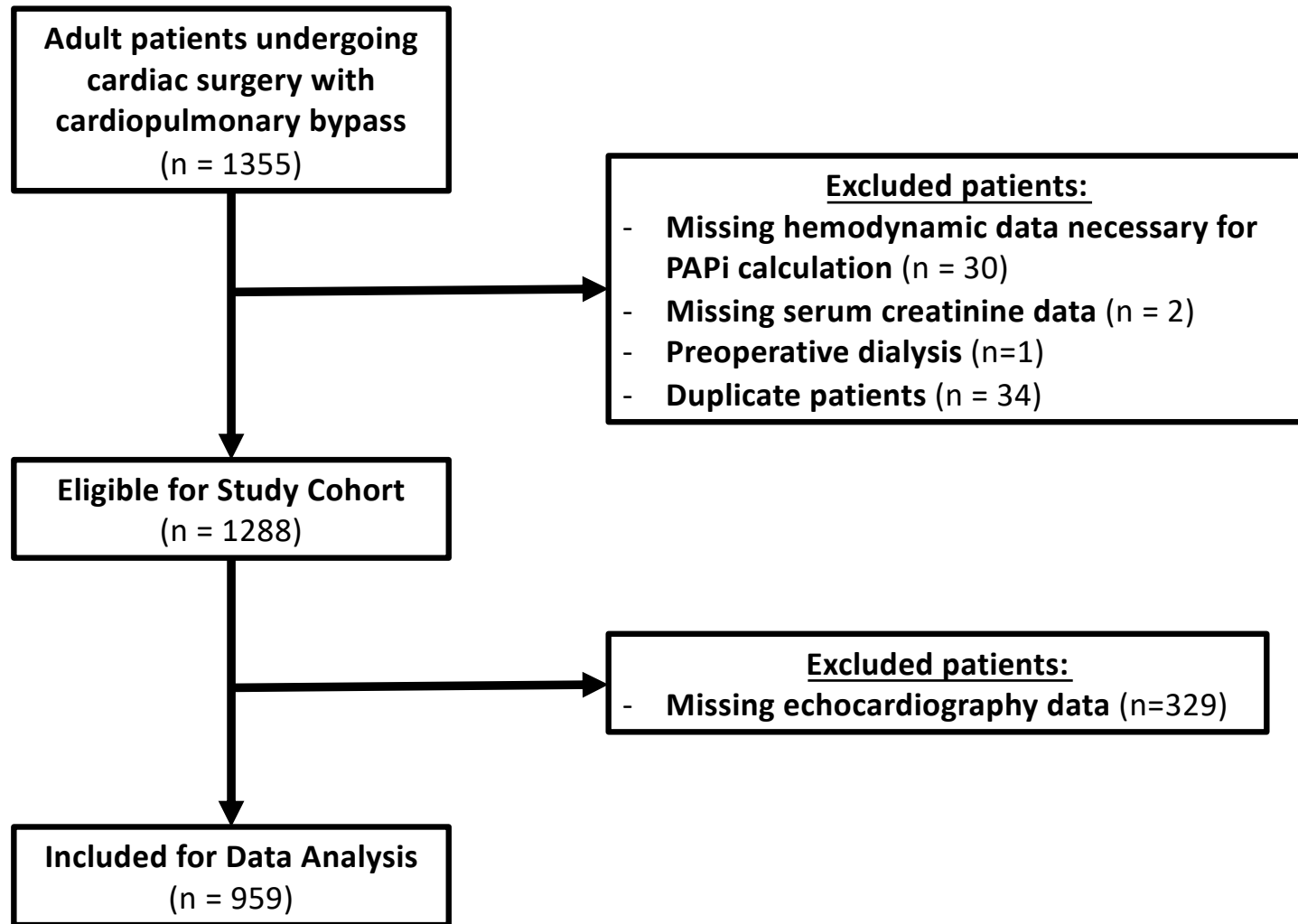

Supplement: Supplementary Materials — Supplementary Figure 1: flowchart of study design with reasons for exclusion. Supplementary Figure 2: covariate balance after propensity-score matching for PAPi > 2.0 using selected variables. Dark bars represent a standardized mean difference (SMD) of 0.2 and dashed bars represent an SMD of 0.1. Supplementary Figure 3: strip plot depicting the distribution of nonmissing values for cardiac index (blue) with imputed values (red). 25 imputations of 10 iterations each were performed. The final estimates used were pooled from imputed values. Supplementary Table 1: multivariable regression table with % ΔPAPi as an outcome of interest, with all included predictor variables. Supplementary Table 2: sensitivity analysis of multivariable regression analysis using imputed values for hemodynamic data and imputed values adjusted by ±10% and ±20% of nonmissing values for cardiac index. [file 5408008.f1.zip › suppfig1.pdf]

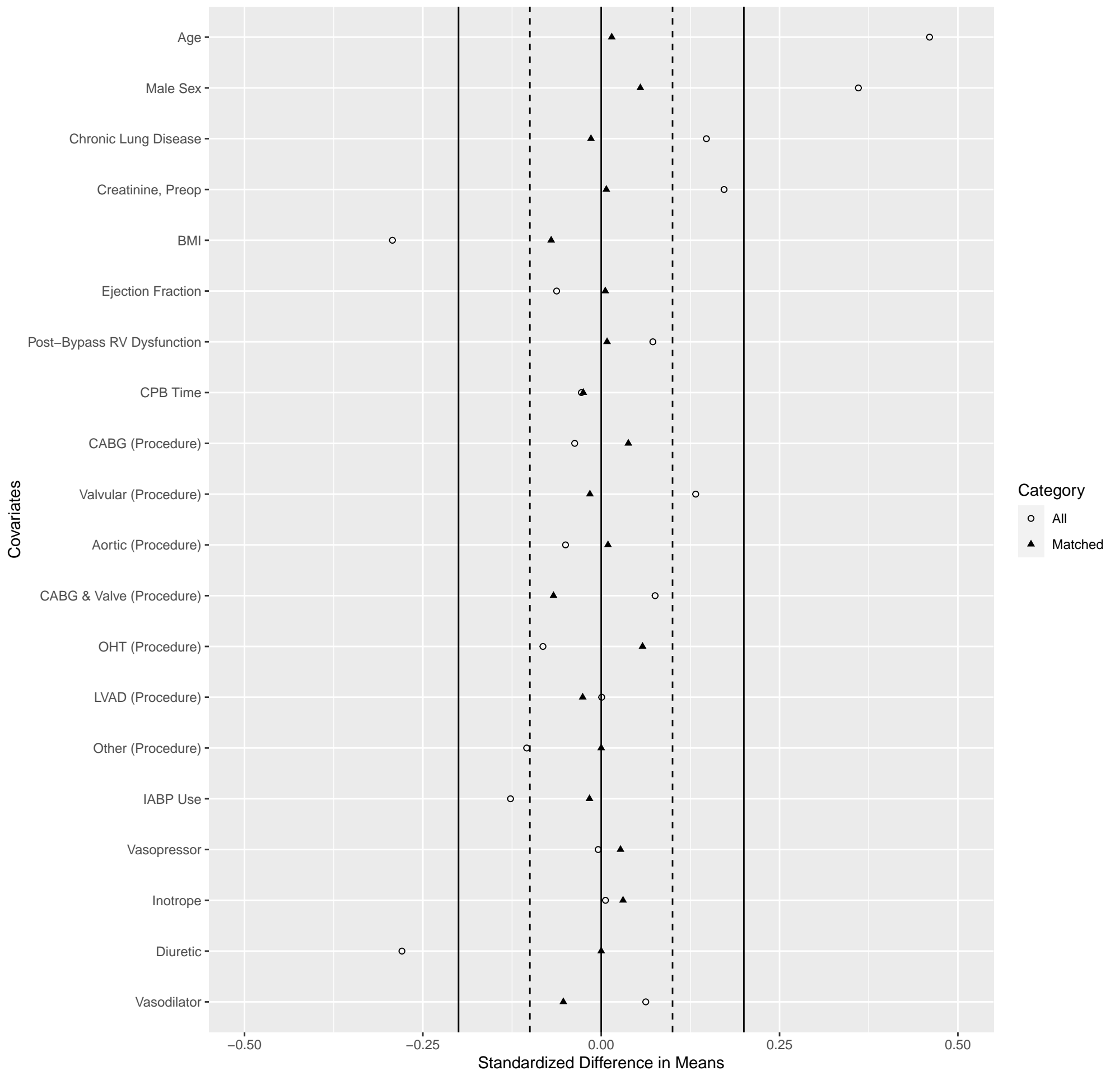

Supplement: Supplementary Materials — Supplementary Figure 1: flowchart of study design with reasons for exclusion. Supplementary Figure 2: covariate balance after propensity-score matching for PAPi > 2.0 using selected variables. Dark bars represent a standardized mean difference (SMD) of 0.2 and dashed bars represent an SMD of 0.1. Supplementary Figure 3: strip plot depicting the distribution of nonmissing values for cardiac index (blue) with imputed values (red). 25 imputations of 10 iterations each were performed. The final estimates used were pooled from imputed values. Supplementary Table 1: multivariable regression table with % ΔPAPi as an outcome of interest, with all included predictor variables. Supplementary Table 2: sensitivity analysis of multivariable regression analysis using imputed values for hemodynamic data and imputed values adjusted by ±10% and ±20% of nonmissing values for cardiac index. [file 5408008.f1.zip › suppfig2.pdf]

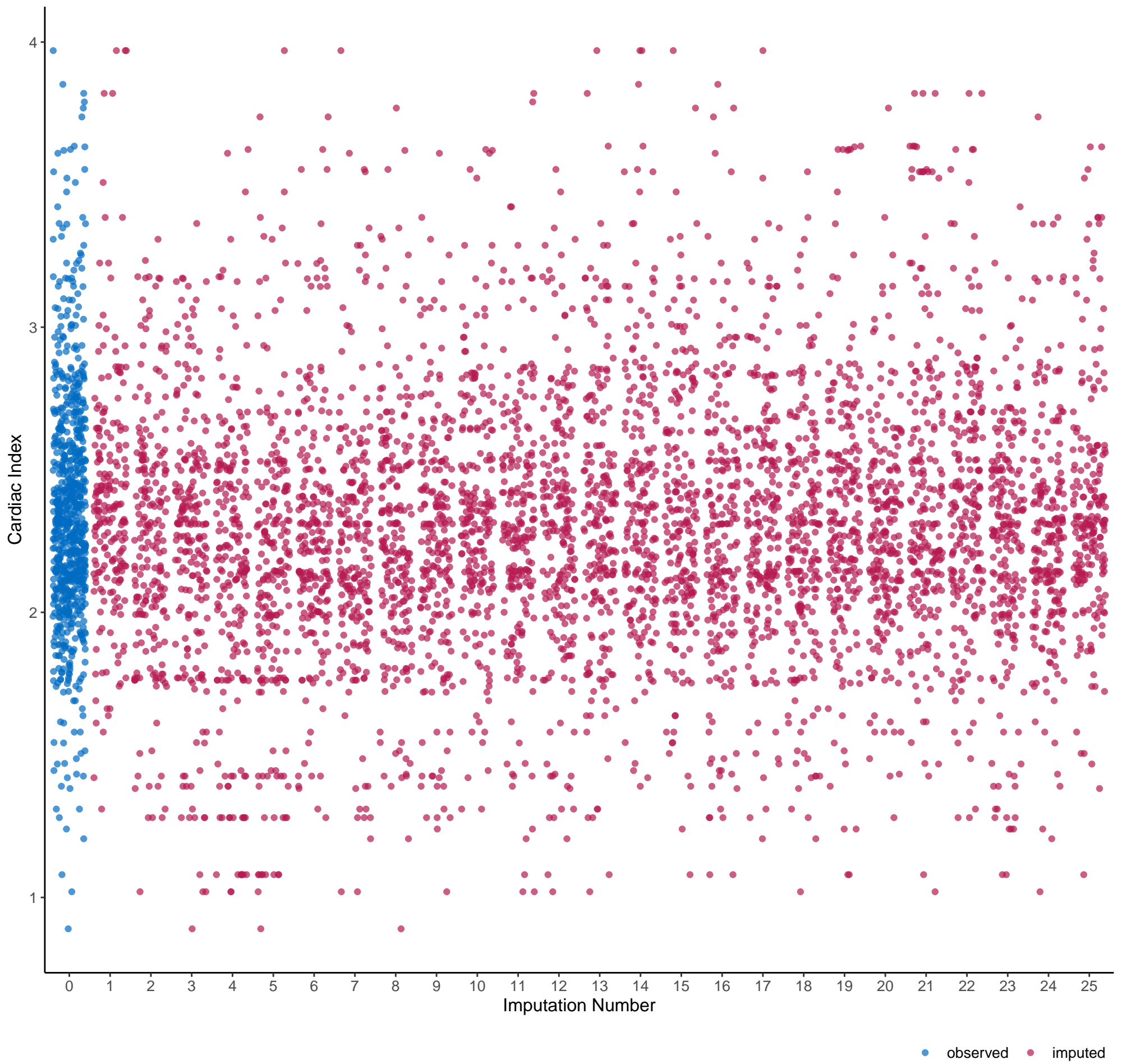

Supplement: Supplementary Materials — Supplementary Figure 1: flowchart of study design with reasons for exclusion. Supplementary Figure 2: covariate balance after propensity-score matching for PAPi > 2.0 using selected variables. Dark bars represent a standardized mean difference (SMD) of 0.2 and dashed bars represent an SMD of 0.1. Supplementary Figure 3: strip plot depicting the distribution of nonmissing values for cardiac index (blue) with imputed values (red). 25 imputations of 10 iterations each were performed. The final estimates used were pooled from imputed values. Supplementary Table 1: multivariable regression table with % ΔPAPi as an outcome of interest, with all included predictor variables. Supplementary Table 2: sensitivity analysis of multivariable regression analysis using imputed values for hemodynamic data and imputed values adjusted by ±10% and ±20% of nonmissing values for cardiac index. [file 5408008.f1.zip › suppfig3.pdf]
